# “Helpful in Thinking Through the Question”: Redesigning the Patient Health Questionnaire-8 for Depression Assessment to Enhance Patient Engagement and Understanding

**DOI:** 10.2196/76230

**Published:** 2026-04-21

**Authors:** Hannah Dorris, Jenna Sheldon, Courtney Sepucha, Emmanuel Kasuti Makau, Pape Sow Traoré, Elizabeth Murnane, Nicholas Jacobson

**Affiliations:** 1 Center for Technology and Behavioral Health, Dartmouth College, 46 Centerra Parkway, EverGreen Building, Lebanon, NH, 03766, United States, 1 7202894016; 2 Thayer School of Engineering, Dartmouth College, Hanover, NH, United States; 3 Department of Computer Science, Dartmouth College, Hanover, NH, United States

**Keywords:** Patient Health Questionnaire-8, PHQ-8, survey design, depression, human-computer interaction, user experience

## Abstract

**Background:**

Major depressive disorder (MDD) is a prevalent and impairing mental health condition characterized by persistent low mood and diminished energy. Self-report instruments, such as the Patient Health Questionnaire-8 (PHQ-8), are frequently used in clinical and research settings for depression assessment.

**Objective:**

We developed, redesigned, and tested 2 alternative versions of the PHQ-8 that incorporate visual elements and reframed statements aimed at improving acceptability and comprehension.

**Methods:**

In a mixed methods study, 20 participants with high subclinical to moderately severe depressive symptoms provided feedback after completing 3 versions: the standard PHQ-8 form, our version augmented with visual aids, and our version that combined visual aids with rephrased item statements for greater clarity and engagement.

**Results:**

Eighty percent (16/20) of participants preferred one of the augmented PHQ-8 versions over the standard questionnaire (*P*=.006).

**Conclusions:**

Participants reported that the augmented versions improved their understanding of survey items, reduced response burden, and increased engagement, suggesting that visual and linguistic design modifications may enhance the patient experience in depression screening.

## Introduction

### Background

Clinicians and researchers distribute health questionnaires every day across a variety of media, such as phones, tablets, computers, or paper forms in clinical waiting rooms. Although these instruments are ubiquitous, patients often report challenges related to engagement and ease of use, motivating design research aimed at improving respondent experience and potentially supporting more consistent reporting behaviors [1].

Psychiatrists and psychologists often heavily rely on the Patient Health Questionnaire (PHQ)-8 (or PHQ-9, which includes an additional item assessing suicidal ideation) to assess major depressive disorder (MDD) severity [2-8]. In this paper, we aimed to explore the redesign of the PHQ-8 by enhancing question framing and language and adding metaphor-based visualizations. Specifically, we created 2 new questionnaires to address limitations of the standard PHQ-8 version, one augmented with visual aids and another that combined visual aids with rephrased item statements to provide greater clarity and engagement. We tested these 2 new versions with participants with depressive symptoms to evaluate the effectiveness of the new designs in terms of patient experience.

We aimed to examine the following research questions:

- How do our redesigned questionnaires, augmented with visuals and other elements, effectively address the limitations of the current PHQ-8?
- Do the augmented PHQ-8 questionnaires show acceptability when tested?
- What contributes to patient preferences for the standard or the augmented PHQ-8 questionnaires?

While design adaptations cannot eliminate all limitations common to mental health screening tools, they may improve user experience, comprehension, and perceived engagement.

### Related Work

#### Overview

Our research is situated at the intersection of the clinical and human-computer interaction literatures. Specifically, the following subsections review how conventional questionnaires often fail to adequately engage patients and accurately capture subjective mental health experiences, the influence of mood symptoms and perceptions of stigma on self-reporting practices, and design strategies for enhancing patient engagement and understanding.

#### Challenges in Self-Report Questionnaires and Known PHQ-8 Biases

We first broadly examine issues with participant burden and engagement in filling out patient questionnaires before examining research on the PHQ-8 specifically. Self-report questionnaires collect important subjective data and are the gold standard for understanding a patient’s condition, particularly for mental health conditions. However, questionnaires can be difficult and burdensome to fill out, especially when a person is busy [9,10]. Research shows that patients generally have low questionnaire response rates, provide careless responses, and struggle with health questionnaire literacy [11-17]. These challenges reflect the general properties of self-report instruments rather than limitations unique to the PHQ-8.

Prior work across diverse clinical populations, including hospice and youth mental health settings, has shown that patients often perceive standardized questionnaires as impersonal or insufficiently reflective of their lived experiences, which can reduce engagement [18,19]. Although these findings are not specific to depression screening, they illustrate broader challenges in designing self-report tools that feel meaningful, comprehensible, and engaging to respondents. These findings highlight a widespread need among patients in mental health, hospice, and general care for improved questionnaire design that minimizes boredom and fosters a greater sense of being understood.

Prior research has demonstrated that the PHQ-8 is subject to several areas of bias that differ from broad research on survey engagement. The main areas highlighted in prior research are recall bias, social desirability bias, and context-dependent comprehension issues. First, in exploring recall bias, recent research has demonstrated that PHQ-8 results have “modest peak-end recall bias” when tested in that the most intense and proximate emotion disproportionately affects the recall and reporting of symptoms [20]. These findings were further examined in another study that demonstrated fluctuations in patients’ ability to recall symptoms, although the recall bias was not statistically significant [21]. PHQ-8 responses may also be influenced by social desirability bias.

Although social desirability bias and recall bias both impact the PHQ-8, comprehension issues are another element that is critical to examine. For example, a recent study found that when tested in another context, there were incorrect interpretations of half of PHQ-8 items and, specifically, issues in interpreting the time scale aspect of the rating [22,23]. Such misunderstandings may partially contribute to discrepancies occasionally observed between clinician-rated and self-rated depression severity [24]. Overall, many of these challenges (eg, recall bias, response burden, and social desirability) are common across screening instruments and modes of administration; thus, design improvements may help mitigate but not fully eliminate them.

#### Mood Questionnaires Need to Take Into Account Common Symptoms

Anhedonia is a common symptom in MDD, where patients experience a loss of interest, motivation, engagement, and inability to experience pleasure. Patients often describe anhedonia as involving not only reduced interest but also a broader flattening of emotion and diminished capacity for joy [25,26]. Therefore, when questionnaires are boring and unengaging, they can be particularly challenging for such patients to complete, compromising response rates and accuracy.

Another major component of depressive symptoms relates to perceived stigma. Stigma is known to interfere, at minimum, with how individuals choose to self-identify in having a mental illness, such as perceived need and help-seeking behavior [27,28]. Research has begun to address minimizing stigma-based bias in questionnaire design, particularly with respect to reducing social desirability bias [29]. Overall, considering 2 commonly occurring depression symptoms are anhedonia and stigma, it is important to work to understand ways to further increase participant engagement and reduce social desirability bias in the PHQ-8 survey for patients with MDD. These factors underscore the importance of designing depression assessments that minimize perceived judgment and support honest self-disclosure.

#### The PHQ-8 Might Have a Familiarity Bias

The PHQ-8 is the most popular questionnaire for assessing depression [30-34]. Recent studies have begun to untangle the relationship between reaction time and PHQ-8 results in individuals with depression, those with depression not seeking treatment, and those with depression seeking treatment. There was a notable difference in reaction time, as well as other observed psychometric properties, in patients with MDD completing questions 3, 7, 8, and 9 of the PHQ-8 more quickly between those who sought treatment and those who did not. These differences indicate the potential for familiarity bias to affect specific questions of the PHQ-8, yet no large empirical study has tested this claim further [35].

#### Designing for Patient Engagement and Understanding

We hypothesize that reasons for the PHQ-8 questionnaire failing to accurately capture MDD symptoms may be due to the instrument’s structure and presentation of questions, particularly in terms of how both its visuals and texts could better promote engagement and motivation.

Visual-based design methods, such as interactive formats and adaptive questions, have begun to be implemented in surveys to enhance patient engagement. Broadly, there has been a rise in using design methods to encourage patient acceptance and understanding across a number of digital platforms, such as conversational agents, collaborative care, vital monitoring, and online cognitive behavioral therapy training [36-42]. For example, a chronic pain survey was developed in which patients select facial expressions to self-report pain levels. The study found high validity in this measure and that patients reported lower response burden and increased response motivation [36].

Other visual designs have explored the use of photos to measure affect, particularly the Photographic Affect Meter interface that asked respondents to select from a variety of pictures to choose the image that best represented their current mood. Evaluation of the Photographic Affect Meter instrument has shown strong validity and ease of use [43]. A similar photo-based questionnaire has been created to measure current pain levels [36]. Collectively, these studies demonstrate that visual and interactive elements can improve usability, reduce response burden, and increase engagement across various health contexts [36,43,44].

Visual designs based on the connection of emojis and mood have also been implemented. Studies on emoji-based tools, such as the EmojiGrid and the Emoji Current Mood and Experience Scale, highlight the potential of visual surveys to be both engaging and valid across a variety of settings [45,46]. However, emojis may be less effective in capturing nuanced emotional states, particularly among individuals experiencing high emotional intensity [47].

Many other studies have found that visual interface elements increase perceived usability and enjoyment and help patients feel more relaxed and happier [48,49]. Furthermore, research finds that increased visual appeal can lead to increased trustworthiness [50]. Considering that patients with mental health concerns often face a sense of stigma when completing assessments [18], visual elements may be an important way to build engagement and trust and elicit more honest responses. Our research, therefore, aims to overcome PHQ-8 challenges related to boredom, stigma, and inaccurate responses by exploring the design of a questionnaire that incorporates visual elements to enhance respondent user experience and engagement.

In addition to visual design, the phrasing and structure of questionnaire items also influence respondent engagement and comprehension. Text-based design is equally important, as the language and framing of items can substantially influence survey experience and response quality. In particular, it is helpful to frame survey items as a question rather than as a statement, as the PHQ-8 currently does [51]. Such framings can also impact how comfortable respondents feel in sharing negative responses [52,53]. Open-ended questions are another effective technique to encourage patient comfort and honesty, as such formats allow for more authentic feedback [54]. A hybrid approach of including a Likert scale with an open-ended question can enhance patient comfort and engagement even without the need for artificial intelligence or large language models to appropriately scale the response [54]. Overall, prior work indicates that incorporating language that is compassionate and open-ended can help encourage respondent engagement and honesty [55-57]. We therefore experimented with modifying the PHQ assessment to evaluate whether such question framings could enhance engagement.

Despite extensive research on self-report burden, visual communication, and item design across health questionnaires, relatively little work has examined how these principles can be applied to improve the PHQ-8 itself. Existing studies reveal important limitations such as recall biases, social desirability influences, and comprehension challenges, but these issues are typically reported at the level of general self-report methodology rather than addressed through targeted redesign of PHQ-8 items or layout. Furthermore, while visual and interactive elements have shown promise in enhancing engagement in other symptom-tracking tools, their effects on depression screening instruments remain largely unexplored, and prior work has rarely assessed whether such design changes preserve psychometric properties. Taken together, this literature highlights a clear gap: the need for systematically designed and evaluated adaptations of the PHQ-8 that incorporate visual elements and improved item phrasing to support patient engagement and understanding without compromising validity. Our study addresses this gap by developing and testing 2 redesigned PHQ-8 formats that integrate these design principles.

## Methods

### Participants

Participants were recruited from a larger Dartmouth study that examined digital treatments for anxiety and depression. Participants were recruited from across the United States via Google and Meta Ads. Individuals had previously completed the PHQ-8 and General Anxiety Disorder-7 screening questionnaires and were eligible for recontact if they were aged between 40 and 65 years, lived in the United States, and demonstrated high subclinical to moderately severe depressive symptoms (PHQ-8≥8). From this pool, we recruited 20 participants (women: n=16, 80%; and men: n=4, 20%). Their mean PHQ-8 score at screening was 14.84 (SD 5.18), which is approximately 5 points above the clinical threshold of 10. Three participants scored below the clinical threshold of 10.

### Data Collection

Each participant completed a semistructured interview via Zoom lasting 30 to 60 minutes. Interview topics included condition acceptance, social support, mindset, stigma, and technology preferences. Specifically, the questions most salient to this study were as follows: At what age did your depression begin? How many years have you been living with depression? Are you currently seeking treatment? Is there any treatment or medication you’ve done to help your condition in the past?

**Figure 1.**
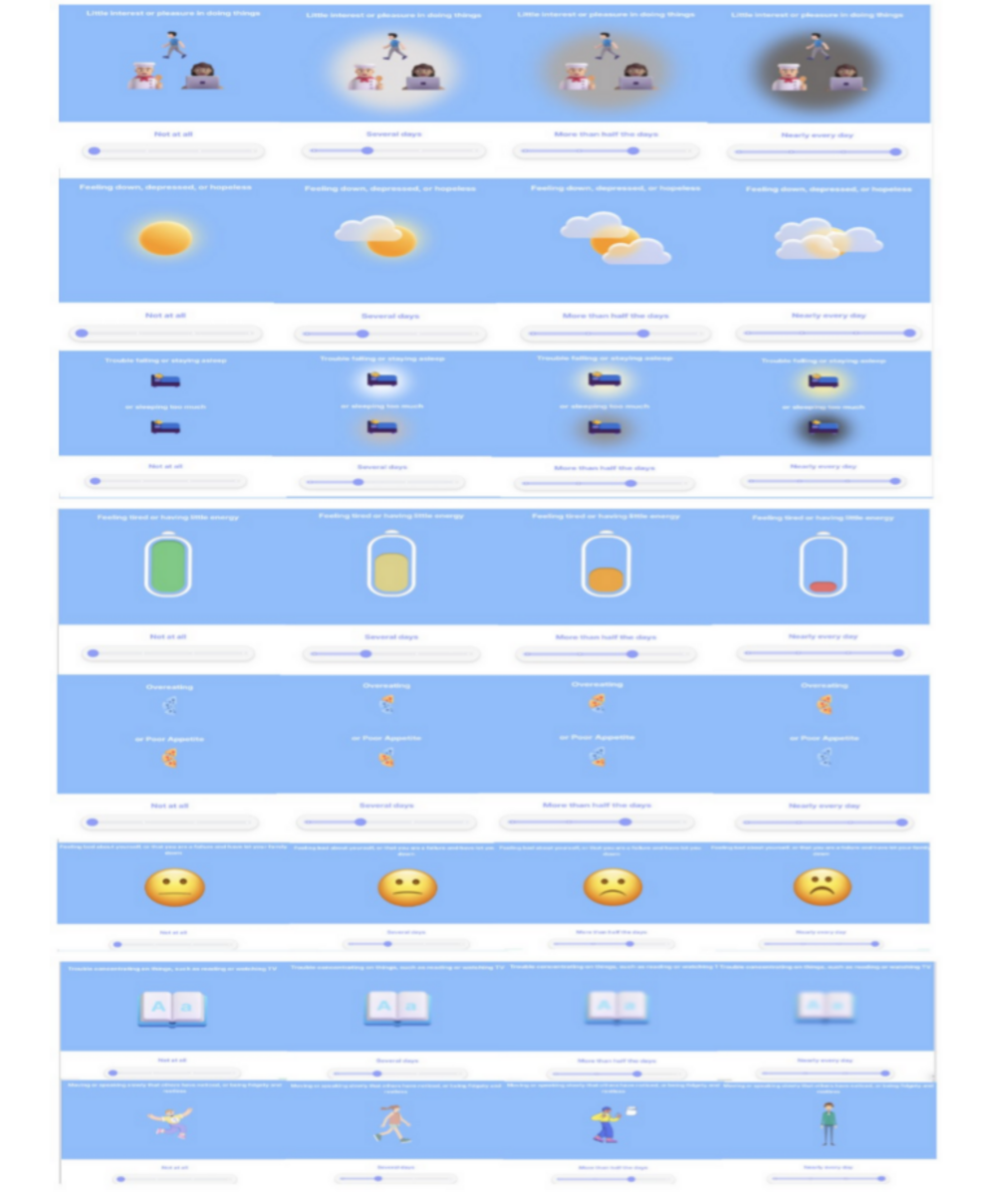
Our visual Patient Health Questionnaire-8 design that augments the standard questionnaire with visual aids.

**Figure 2.**
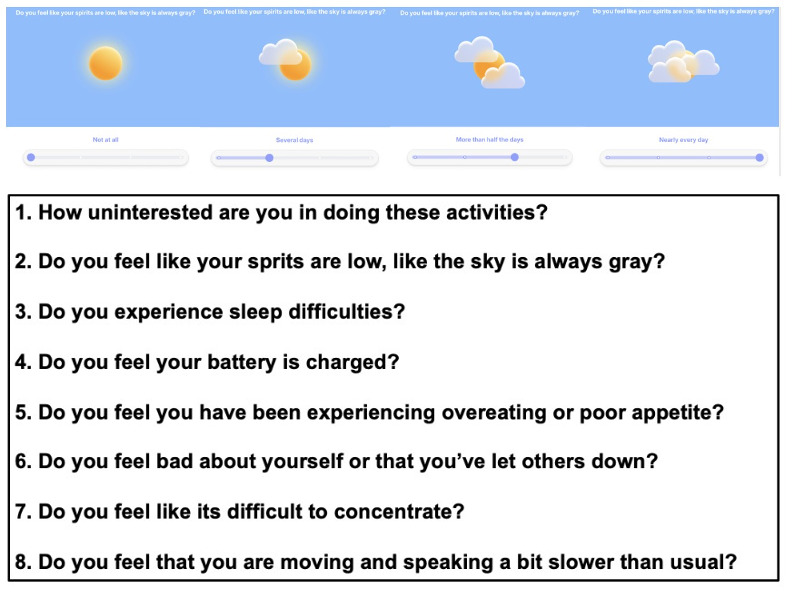
Our reframed Patient Health Questionnaire-8 design that both augments the standard questionnaire with visual aids and also rephrases survey items.

Following the interview, participants were given 2 PHQ-8, as shown in Figure 1 (we did not include the 9th question regarding suicidality, as we had already screened for this, as described earlier). The “Visual PHQ-8” (Figure 1) used the same statements of the standard PHQ-8 accompanied by visualizations. The “Reframed PHQ-8” (Figure 2) modified the assessment statements to open-ended questions, while using the same visualizations from our Visual PHQ-8. We rephrased all the PHQ-8 items to be open-ended and more conversational. All the questions besides item 1 reflect concrete feelings or behaviors, whereas item 1 reflects a motivational state (“little interest or pleasure in doing things”). Using a “how” structure helps preserve that motivational-state meaning, while a “do you...” phrasing would incorrectly frame the item as a feeling or behavior rather than a motivational state. After completing each questionnaire, we asked participants about their experience in filling out the questionnaire and whether there was anything they would change about it. Finally, we asked participants if they had a preference among the various PHQ versions (standard, visual, and reframed) and to elaborate on such reactions. To enable us to examine links between survey preference and psychological status, participants were then given the following questionnaires: Interpersonal Social Support to capture dimensions of social support (eg, tangible, appraisal, and belonging) [58,59]; Trait Hope Scale [60,61], which is known to predict positive outcomes and resiliency; Kind of Person Scale [62,63] and Dweck Growth Mindset Scale [64] to assess growth mindset and reactions to adversity; and the Stigma Scale for Chronic Illnesses to assess psychological distress and patient performance [65].

### Data Analysis

Interviews were transcribed and coded using an inductive qualitative approach. Two researchers independently reviewed all transcripts, developed an initial codebook, and resolved discrepancies through consensus. Themes were organized around depression history, current treatment status (ie, whether an individual was actively seeking therapy or medication), and questionnaire preferences.

We conducted 1-tailed exact binomial tests to evaluate whether the proportion of participants preferring augmented PHQ versions significantly exceeded chance (50%). In addition, we analyzed participants’ responses to each of the PHQ-8 questionnaires. Specifically, we computed means and SDs for each questionnaire group and conducted intraclass correlation coefficients (ICCs) between the questionnaires. Finally, we analyzed responses to the Interpersonal Social Support [58,59], Trait Hope Scale [60,61], Kind of Person Scale [62,63], Dweck Growth Mindset Scale [64], and the Stigma Scale for Chronic Illnesses [65] to identify associations between those responses and participants’ preferences among the different PHQ-8 versions.

### Ethical Considerations

The study conducted at Dartmouth was approved by the Committee for Protection of Human Subjects under study 32782. All activities in this study were conducted under the approved protocol and complied with institutional and federal guidelines for research on human participants. All participants gave informed consent and were compensated ($20 per hour) at the completion of the study. We attest to maintaining privacy and confidentiality of all research subjects’ data.

## Results

### Overview

**Figure 3.**
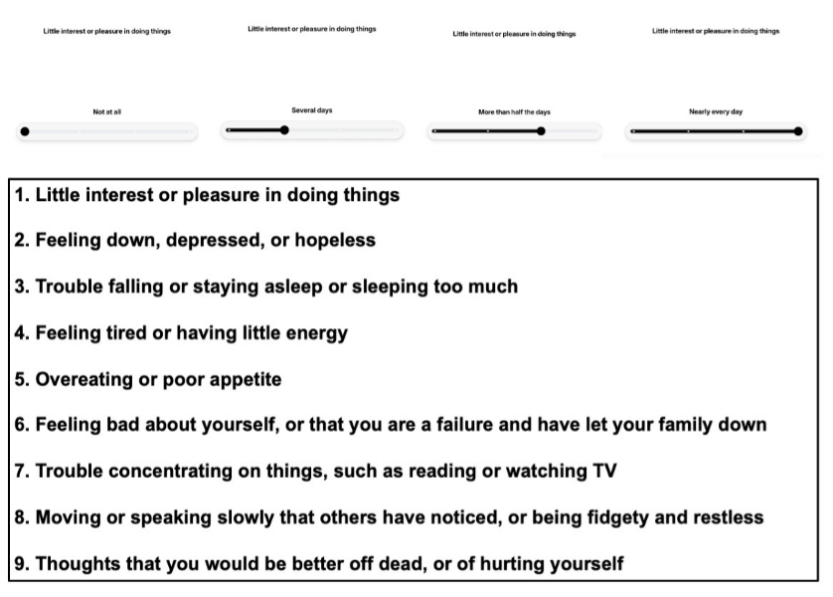
The Patient Health Questionnaire-8 in standard form.

Our data showed that 80% (16/20) of participants preferred one of the redesigned PHQ-8 formats over the standard version. This preference for an augmented version of the PHQ-8 was statistically significant under a 1-tailed exact binomial test (proportion: 0.80, 95% CI 0.56-0.94; *P*=.006), indicating an above-chance likelihood that a participant prefers a redesigned PHQ-8 (Figure 1) compared to a traditional PHQ-8 (Figure 3, Tables1  2). Only 20% (proportion: 0.20, 95% CI 0.06-0.44) of participants preferred the standard PHQ-8, indicating a minority preference for the nonaugmented version.

Participants who preferred the redesigned questionnaire with visualizations (PHQ-8 mean 12.50, SD 5.34) had higher PHQ-8 scores than participants who preferred the traditional statements (PHQ-8 mean 9.75, SD 2.99). Additionally, the trend followed that those who preferred visualizations over statements had higher stigma scores (mean 23.19, SD 9.63) and lower hope scores (mean 40.94, SD 8.64) than those who preferred statements (stigma: mean 21.25, SD 10.11; and hope: mean 51, SD 6.58).

**Table 1.** Patient Health Questionnaire (PHQ) responses, summarized according to survey type preference.

Participants’ favorite questionnaire	Count (N=20), n (%)	PHQ-8: statements (mean 11.95), mean (SD)	PHQ-8: statements and visualizations (mean 11.00), mean (SD)	PHQ-8: statements and questions (mean 10.60), mean (SD)	Trait hope scale (mean 42.95), mean (SD)	Stigma scale (mean 22.80), mean (SD)	Growth mindset (mean 10.11), mean (SD)	Kind of person (mean 28.50), mean
Prefer statements	4 (20)	9.75 (2.99)	6.75 (4.86)	6.75 (2.06)	51.00 (6.58)	21.25 (10.11)	10.50 (6.24)	28.00 (4.08)
Prefer questions and visualizations	7 (35)	11.29 (5.53)	10.71 (5.02)	10.43 (4.58)	38.14 (12.13)	25.29 (9.93)	11.14 (5.01)	27.43 (7.21)
Prefer statements and visualizations	9 (45)	13.44 (5.32)	13.11 (5.75)	12.44 (6.48)	43.11 (4.17)	21.56 (9.66)	9.00 (3.38)	29.56 (3.78)
Prefer either questions or statements and visualizations	16 (80)	12.50 (5.34)	12.06 (5.41)	11.56 (5.64)	40.94 (8.64)	23.19 (9.63)	10.00 (4.21)	28.63 (5.44)

**Table 2.** Response per question for the 3 Patient Health Questionnaire-8 (PHQ-8) versions.

Questionnaire type	Anhedonia, mean (SD)	Hopelessness, mean (SD)	Sleep, mean (SD)	Fatigue, mean (SD)	Appetite, mean (SD)	Self-esteem, mean (SD)	Concentration, mean (SD)	Movement, mean (SD)
PHQ-8: statements	1.75 (1.00)	1.85 (1.03)	2.00 (0.97)	2.10 (0.91)	1.15 (1.12)	1.25 (0.85)	1.35 (1.05)	0.55 (0.84)
PHQ-8: statements+visualizations score	1.45 (1.09)	1.55 (1.05)	2.05 (0.94)	1.95 (1.05)	1.00 (1.12)	1.10 (0.64)	1.35 (1.14)	0.50 (0.83)
PHQ-8: questions+visualizations score	1.30 (0.97)	1.40 (1.05)	2.00 (1.17)	1.55 (1.32)	1.00 (1.08)	1.42 (0.96)	1.40 (1.14)	0.55 (1.00)

We next discussed the differences between the augmented versions (ie, questions and visualizations vs statements and visualizations) descriptively, as the primary statistical analysis evaluated whether participants preferred an augmented PHQ-8 compared to the standard version. We only use descriptives in discussing the differences between the augmented versions, as both augmented conditions incorporated visualizations and the study design did not include a condition with just rephrased questions, limiting the ability to generalize and analyze the effect of question phrasing independently. We now specifically examine the adapted versions, where 45% (9/20) of participants (proportion: 0.45, 95% CI 0.23-0.68) preferred the visual-only PHQ-8, and 35% (7/20) of participants (proportion: 0.35, 95% CI 0.15-0.59) preferred the version that combined visuals with reframed questions. The 95% CI for each of the augmented versions demonstrates a similar spread of preference for the questions and visualizations and statements and visualizations, which could be further studied in future experiments.

### Participants Who Preferred Visualizations

Participants who preferred the addition of visualizations (Figure 1) strongly emphasized that they felt more engaged and experienced more pleasure while taking the questionnaire. For example:

It was more engaging and fun...I really liked that. I liked having the visual.

[Participant 18]

I did find it more fun.

[Participant 19]

I liked the little emojis. That was sort of fun.

[Participant 17]

Importantly, we identified that the visualizations not only were more engaging but also prompted more reflective responding. For example, participants noted that:

It would make me stop and think. About things that I generally don’t.

[Participant 1]

I think some of the visuals were helpful in thinking through the question.

[Participant 11]

Both these participants noted that having the visual helped them pause and think more deeply about the question.

### Participants Who Preferred Visualizations and Questions

Participants who had a preference for the questionnaire that included both visualizations and reframed questions (Figure 2) explained that this design helped them feel both cared about and understood. Furthermore, participants discussed that the wording felt less negative:

I liked the way that they were worded. Like they just seem softer than the normal [instrument phrasings].

[Participant 6]

Similarly, other participants expressed that the reframed questions felt less negative and confrontational, and more compassionate and genuine in checking in on a person’s status. For example:

The questions were phrased differently, and I liked that. It didn’t feel negative. It was more like, “oh, we would like to know this,” rather than “we know you’re like this, just admit it.”

[Participant 9]

This theme related to a sense of being cared about and understood was further elaborated by 2 other participants:

Yeah, I like these questions better...They speak to you a little bit more.

[Participant 12]

It felt more understanding, and it felt like whoever was at the other end of the survey really wanted to know to help, rather than really wanting to know because it will be useful to them.

[Participant 14]

The second common theme was that participants felt that the reframed questions enabled them to be more thoughtful in responses. For example:

They made me think more... It’s getting at the same information, but I like the way it was worded. It is a more thoughtful way, I think.

[Participant 6]

Another participant echoed this statement, noting that:

It made me think because it doesn’t phrase it the same way that the doctors do. I had to think about what are you asking.

[Participant 9]

The ability of the redesigned questions to be more thought-provoking may partly stem from providing a break from the repetitive nature of the traditional PHQ-8 statements:

I’m so used to those questions I just kind of speed through them.

[Participant 6]

...they get so repetitive. I’ve done where you track it; it just gets really like, “okay, I was depressed yesterday, I’m depressed today...

[Participant 9]

In this way, participants emphasized that the redesigned questions helped overcome the repetitive nature of the PHQ-8 and, in turn, helped them to be more thoughtful in their responses.

Participants also explained the redesigned questions and visuals also supported better item comprehension:

The questions were a lot better than the statements...Sometimes with the statements, it’s difficult to put together what it actually means.

[Participant 16]

The cloud and gray sky [visualization and question], I was like, “oh, I can imagine that.”

[Participant 19]

Overall, while these participants explained that they felt that responses to the traditional PHQ-8 were “difficult to put together,” they expressed that new visualizations helped them “imagine” the statements better.

### Participants Who Preferred Statements

Despite most participants preferring redesigned versions of the PHQ-8, 20% (4/20) of participants had a preference for keeping the PHQ-8 the same with no addition of a visualization or reframed question. We observed 2 main themes for these individuals. First, they found the addition of the visualizations to be hard to relate to. For example:

The visualizations are distracting because it’s somebody else’s picture*.*

[Participant 3]

This participant and another also noted their distaste for the aesthetics of the visualizations, referring to them as childish:

they seemed kind of child-like.

[Participant 3]

I think the text is enough. [Better than the] stupid smiley face chart they have at the hospitals and doctors’ offices.

[Participant 3]

Another theme that we observed was that the visualizations were seen as unnecessarily lengthening the self-report process or generally not necessary given how familiar participants were with the standard PHQ-8:

Honestly, I don’t really pay attention to the visuals, so I don’t think they’re necessary*.*

[Participant 3]

They later explained that just having the statement was sufficient and efficient:

it just makes it shorter, sweeter, faster, less to look at, and less to concentrate on.

[Participant 2]

Participant 20 felt similarly as she had regularly taken the PHQ-8 after attending therapy and psychiatry sessions for over 20 years and therefore preferred the traditional format she was so accustomed to.

### Potential Visualization Improvements

We also asked participants to provide feedback on visualizations and share any ideas for potential improvements. Some feedback about the self-report interface would be easily addressable; for example, tweaking the digital survey to automatically shift to the next question could help respondents more smoothly transition from question to question:

Not the visuals, but I wish I didn’t have to press next next next so many times*.*

[Participant 13]

Other feedback applied to specific PHQ-8 questions. For example, in question 7 (“Trouble concentrating on things, such as reading the newspaper or watching television”), our design increasingly blurred out the visual as the scale increased, and one participant (participant 5) expressed that this design was difficult to see given vision issues. Another participant emphasized that they desired a consistent runner emoji for question 8 (participant 17), and another participant requested questions 3 and 5 to be separated out (participant 12).

### Survey Preference and Patient Histories

We observed a tendency for participants who had been more recently diagnosed to have a higher preference for a redesigned PHQ-8 questionnaire. More specifically, participants who had been diagnosed earlier preferred statements (average time since diagnosis 23, SD 17 y), whereas participants who had been diagnosed later preferred visualizations (average time since diagnosis 18, SD 11 y), and the most recently diagnosed participants preferred visualizations and questions (average time since diagnosis 16, SD 7 y). While this effect is not statistically significant when tested with a one-way ANOVA, it suggests that there is a potential trend where individuals with depression may become accustomed to a specific format over time, whereas those more recently diagnosed individuals may face challenges engaging with these conventional self-report instruments. Of participants who preferred the statements format (n=4), 50% (n=2) were not seeking therapy, 25% (n=1) were in therapy and taking antidepressants, and 25% (n=1) were taking antidepressants but not in therapy. On the other hand, of participants who preferred the statements and visualizations format (n=9), 22% (n=2) were not seeking therapy, 33% (n=3) were in therapy and taking antidepressants, 22% (n=2) were in therapy but not taking antidepressants, and 11% (n=1) were taking antidepressants but not in therapy. All participants who preferred the question and visualization (n=7) format of the survey were actively in therapy, and 86% (n=6) were also taking antidepressants.

### Scoring of PHQ-8 Surveys Compared

The PHQ-8 surveys included 8 questions, all on the same scaling of 0 to 3 with a maximum score of 24. In our sample, the average score of the PHQ-8 in standard form (Figure 3) was 11.95 (SD 5.03), 11 (SD 5.62) for our visual PHQ-8 design that augments the standard questionnaire with visual aids (Figure 1), and 10.6 (SD 5.45) for our reframed PHQ-8 (Figure 2). The total score for each PHQ-8 questionnaire showed ICCs that were highly consistent across the 3 surveys (average raters ICC=0.96, 95% CI 0.93-0.98; *P*<.001).

### Individual Questions of PHQ-8 Surveys Compared

**Figure 4.**
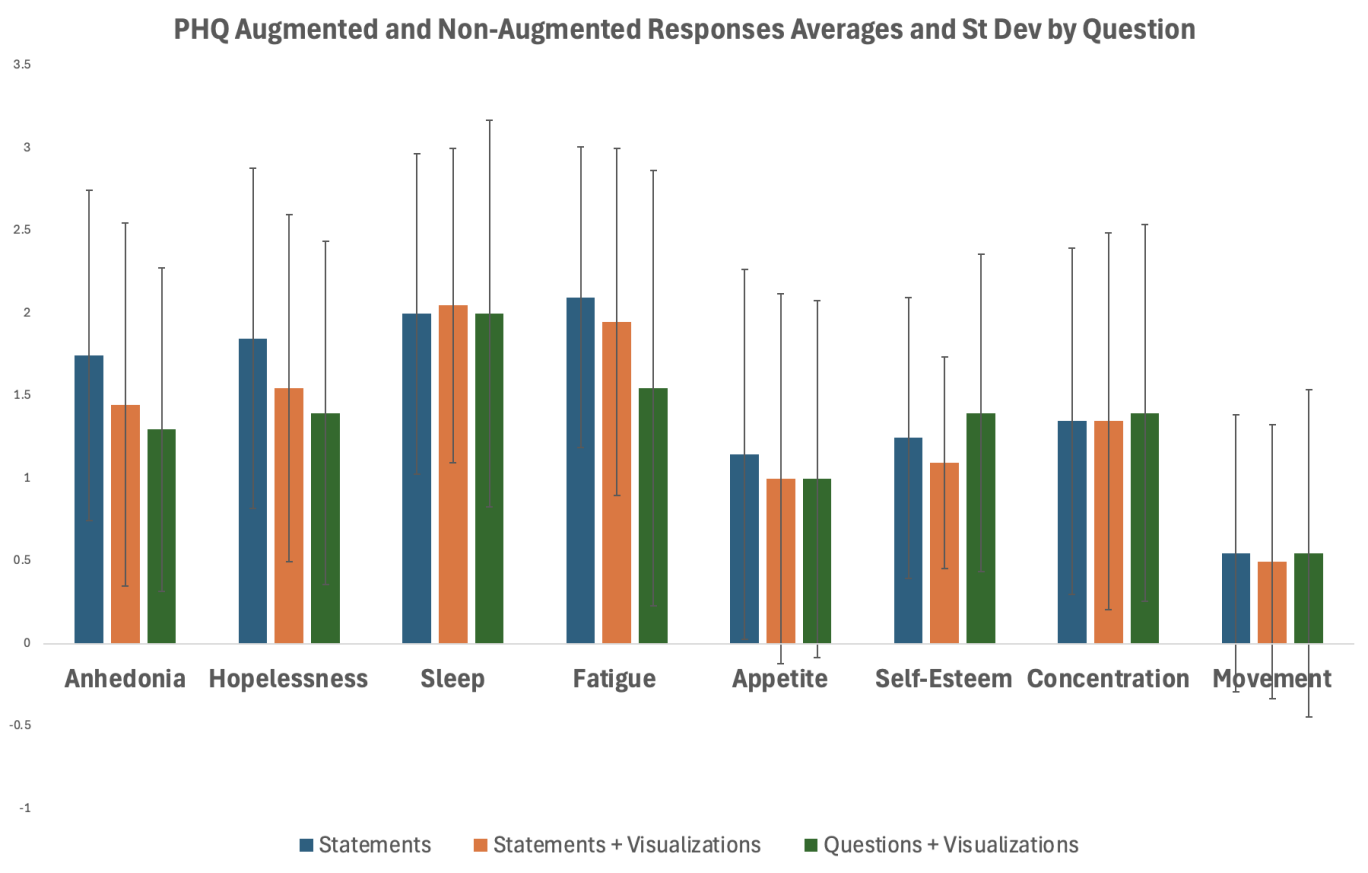
Each of the 3 versions of the Patient Health Questionnaire (PHQ)-8’s individual question responses compared to each other.

Overall, the movement, sleep, concentration, and appetite responses between the 3 versions stayed fairly consistent with the maximum change in averages at 0.15. However, questions related to more visible and stigmatized symptoms seemed to show more variability between the 3 versions, where anhedonia, hopelessness, fatigue, and self-esteem had still low but higher variability maximum change in averages at 0.55 between versions (Figure 4).

The ICC showed high statistical significance across all 8 questions. For anhedonia and fatigue, the ICC estimates ranged from 0.64 to 0.70 (*P*<.001), whereas for hopelessness, sleep, appetite, concentration, and movement, the ICC estimates ranged from 0.88 to 0.96 (*P*<.001). Furthermore, all 8 questions show moderate to high correspondence with the traditional PHQ-8 and statistical significance. This correspondence helps to indicate that the augmented versions are able to effectively measure the psychometric properties assessed in the traditional version of the PHQ-8.

## Discussion

### Principal Findings

This study demonstrates that thoughtfully redesigning PHQ-8 items through the addition of visual elements and conversational reframing may improve people’s subjective experience of completing depression screeners. Across our sample, most participants reported that the redesigned versions felt more engaging, supportive, or easier to interpret compared to the standard PHQ-8. While these adaptations do not change what is being measured, they appear to influence how users approach the task, with some describing the redesigned items as prompting more reflection or making the questions feel more personally meaningful. These findings align with broader work showing that visual and linguistic framing can shape how people make sense of self-report assessments. Specifically, we explored augmented versions of the PHQ-8 instruments, incorporating visualizations and reframed statements aimed at improving respondent engagement and comprehension. Specifically, we examined participant feedback after completing 3 versions of the PHQ-8: the standard form, a version augmented with visual aids, and a version that combined visual aids with item questions rephrased for greater clarity and compassion. We next reflect on the acceptability and overall experience of each of these versions.

### Overwhelming Majority Enjoyed Augmented PHQ-8 Versions

We observed that 80% (16/20) of participants had a significantly greater preference for either augmented version of the PHQ-8 compared to the standard version of the instrument. For the 45% (9/20) of participants who most preferred the version augmented only with visuals, we observed themes related to how these additions promoted higher engagement and thoughtfulness in responses. Although augmented designs for health questionnaires have yet to be tested in the context of depression, previous work that tested the addition of visuals in patient populations such as chronic pain and affect found similar results, suggesting that visualizations may be broadly beneficial. Indeed, the addition of visuals is known to induce greater trustworthiness [50], reduce response burden, and increase response motivation [43,66]. Such findings were mirrored in this study, where patients reported feeling more engaged and thoughtful when using the augmented PHQ-8 with visualizations. Additionally, incorporating visuals into the questionnaires may elicit greater self-reflection, which could influence how participants articulate their internal states in their responses.

For the 35% (7/20) of patients who most preferred the addition of visuals and new question framings, we established a few main themes based on their feedback, which emphasized that these designs helped respondents feel more thoughtful, cared for, and understood. Such findings resonate with prior work where patients were more comfortable, engaged, and authentic in responding to questions rather than statements [51,54-57].

Overall, our findings highlight that survey items augmented with visuals and revised phrasings help patients be more thoughtful while responding, feel more cared for and understood, and generally experience less response burden. An interesting future study, now that this redesign has been piloted, would be to integrate the feedback from our study and test the 3 versions with new patients who are not already so used to the standard format (ie, no change management needed).

### The Minority of Patients Who Enjoyed the Standard Version

Examining the 20% (4/20) of patients who preferred the standard version of the PHQ-8, these participants highlighted that they found the visualizations and question changes to be distracting and that these augmentations lengthened the time it took for them to finish the questionnaire. Their feedback indicated that such reactions may stem from participants feeling a lack of connection to the visual and linguistic changes (eg, not resonating with the metaphor of energy as a battery visualization). Another potential factor we saw was a repetition and familiarity bias, where participants prefer surveys that they are more familiar with [67-69], given many of these participants had been diagnosed earlier and had been using the standard PHQ-8 for a longer number of years. Finally, these participants pointed out how the augmented questionnaire was longer and/or involved more content to consume. While our goal with the augmented questionnaires was to increase engagement and response motivation with these additional visual and textual elements [51,54-57], this feedback is a good reminder that such designs could be perceived as more burdensome or time-consuming for some individuals.

### Patient Preferences in Relation to Demographic Characteristics and Survey Features

We first examined the impressions of those participants who preferred the PHQ-8 augmented with visual aids. These individuals displayed common characteristics in that they were diagnosed with MDD slightly later, on average, than those who preferred the standard statement-based survey. Additionally, more of these participants were actively seeking therapy. As mentioned, we suspect this may be due to less of a repetition bias or familiarity bias that may affect respondents who have a longer history of using the PHQ-8 as part of treatment [67-69]. Furthermore, therapy is known to not only help patients cope with their condition but also to develop greater condition acceptance and both somatic and emotional awareness [70-72]. Participants who are actively seeking therapy may therefore prefer an augmented survey that greater reflects their subjective awareness about condition on both cognitive, emotional, and physical levels.

Further exploring links between patient profiles and survey preferences, we observed a clear trend where participants with a lower depression score, higher trait hope scale, and lower stigma preferred the traditional PHQ-8 statements over the augmented versions. Said another way, as a participant’s levels of depression and stigma increase and hope decreases, there appears to be a stronger preference for augmented PHQ-8 designs (Table 1). We believe this suggests the standard PHQ-8 may be better suited for someone with a more mild condition and that as condition severity worsens, the addition of visualizations and linguistic changes allows for the questionnaire to remain approachable while avoiding perceptions of stigma or judgment [28]. For example, this sense of judgment or marginalization can be seen in an aforementioned quote: “we know you’re like this, just admit it.” Given that prior research has demonstrated the impact that stigma and anhedonia have on survey responses [27-29] and that the current state of the PHQ-8 can fall short in accurately assessing MDD [3,7], it seems that a respondent’s levels of perceived stigma are a key characteristic to understand when determining whether an augmented questionnaire may be more appropriate to deliver.

### Survey Responses

The primary goal of this study was to determine if a PHQ augmented with visual and linguistic elements was acceptable and engaging for individuals with depression. Encouragingly, we found that 80% of participants did enjoy and prefer an augmented questionnaire. We also found that scores were reliable and accurate based on ICCs across all 3 questionnaires (2 augmented questionnaires and the 1 standard PHQ-8 questionnaire). Examining specific questionnaire items, we found that the movement, sleep, concentration, and appetite responses among the 3 versions had the highest agreement, whereas the more visible and stigmatized symptoms of anhedonia, hopelessness, fatigue, and self-esteem had moderate agreement compared to the same items with the traditional PHQ-8. Specifically, participants reported lower scores for these stigmatized symptoms on the PHQ-8 version augmented with visuals. We had hypothesized that our visuals would reduce shame and stigma, leading to higher scores. However, we found that the stigmatized symptom item scores reduced. We hypothesize that the addition of the visuals may have unintentionally softened or reframed the symptoms, which may have led to participants reporting symptoms with less severity. Another potential explanation for the score decrease is that individuals with higher stigma may underreport their symptoms with the addition of the visualizations. Therefore, future work is needed to examine this pattern and unpack how visualizations influence shame, stigma, and self-reporting.

### Limitations and Improvements for Future Research

This study was able to demonstrate broad acceptability and engagement with the redesigned questionnaires; however, it had several limitations that need to be addressed in future studies. First, the sample size was limited to only 20 participants, which limits the generalizability of the study. The study was able to demonstrate that participants engaged with the new material. In future studies, we could further examine test-retest reliability and conduct a large-scale validation study. Additionally, participants completed the questionnaires after an interview about their depression symptoms and symptom management. Although participants’ opinions on the visualizations should likely not have been influenced by this interview, future studies without an interview prior could better control for any possible effect. The study is limited by demonstrating that participants preferred the visual format; however, this study did not test whether this preference accurately corresponds to improved internal validity or accuracy of PHQ-8 ratings. The variation of scores across the formats, although small, indicates that visual elements may influence responses, and therefore, future work would need to ensure psychometric validity of the new measures.

Our sample primarily relied on middle-aged adults (aged 40‐65 y), which could have impacted the relationship with how participants interpreted and connected with the visual aids. Further testing across other age groups could determine if the effects from our study are reproducible age-independent. Additionally, in future studies, increasing the sample size could discern the impact of age and help-seeking behaviors on participants' views on each of the questionnaires and ensure test-retest reliability. One immediate next step is to explore a variety of additional questionnaire refinements based on participant feedback, including, as discussed previously, smoother transitions between questions, keeping visual designs sensitive to differences in people’s visual abilities, using consistent emojis across item scales, and separating out questions that currently mix multiple assessments into one. In these future studies, we also intend to add a fourth survey option, which is to use the same open-ended questions tested in our study but without the addition of the visualizations. The questionnaire will retain its same length yet provide more empathetic and thought-provoking questions. It is necessary to examine more repeated and longitudinal testing of the redesigned instruments to assess their reliability and their user experience over time and in real-world self-report scenarios. Additionally, although the questionnaires had response options that were frequency-based, it is possible that some participants interpreted the visual aids as symptom intensity rather than frequency. It is possible that this juxtaposition could have influenced the interpretation of specific survey items. We intend to further explore how participants interpreted the addition of visuals and if they shifted from frequency to intensity of symptoms in future studies.

### Conclusions

In exploring 2 new PHQ-8 designs that augmented the standard version with visuals as well as reframed item language, we found that participants overwhelmingly supported these augmented PHQ-8 versions. Participants expressed that they felt more engaged, understood, and thoughtful in making self-reports, suggesting that the augmented versions may better capture subjective experience and ultimately provide a more accurate diagnostic assessment. Furthermore, our findings highlight how integrating visual and linguistic modifications into depression screening tools can enhance patient experience without compromising the instrument’s fundamental validity. Overall, this study provides design strategies and preliminary evidence in how to update widely used self-report instruments to be more patient centered.

## Abbreviations

ICC: intraclass correlation coefficient
MDD: major depressive disorder
PHQ-8: Patient Health Questionnaire-8
